# New records of water mites of the family Torrenticolidae (Acari, Hydrachnidia) with descriptions of two new species from Nanshih River system in Taiwan and redescription of *Torrenticola ussuriensis* (Sokolow, 1940) from the Russian Far East

**DOI:** 10.3897/zookeys.116.1253

**Published:** 2011-07-07

**Authors:** Vladimir Pešić, Ksenia A. Semenchenko, Tapas Chatterjee, Rita S.W. Yam, Benny K.K. Chan

**Affiliations:** 1 Department of Biology, University of Montenegro, Cetinjski put b.b., 81000 Podgorica, Serbia and Montenegro; 2 Institute of Biology and Soil Science, Far Eastern Branch of Russian Academy of Sciences, Vladivostok, 690022 Russia; 3 Department of Biology, Indian School of Learning, I.S.M. Annexe, P.O. – I.S.M., Dhanbad-826004, Jharkhand, India; 4 Department of Bioenvironmental Systems Engineering, National Taiwan University, Roosevelt Road, Taipei 10617, Taiwan R.O.C.; 5 Biodiversity Research Center, Academia Sinica, Taipei 115, Taiwan R.O.C.

**Keywords:** Acari, water mites, new species, running waters, Nanshih River system, Taiwan

## Abstract

New records of torrenticolid water mites (Acari: Hydrachnidia, Torrenticolidae) from Nanshih River, Taiwan, are presented. Two new species are described: *Torrenticola nanshihensis* and *Torrenticola taiwanicus*; the latter species is compared with *Torrenticola ussuriensis* (Sokolow, 1940), a poorly known species which is re-described based on a new material from the Russian Far East; *Monatractides* cf. *circuloides* (Halík, 1930)is reported for the first time for Taiwan.

## Introduction

Taiwan, an island situated in East Asia in the Western Pacific Ocean, covering a land area of 35,801 km2, is located off the southeastern coast of mainland China.

The water mite fauna of Taiwan is very incompletely known. History of water mite research of Taiwan started in the beginning of the last century when Lohmann (1909) described a new species of marine water mite genus *Pontarachna* (*Pontarachna formosa*). Later on, Uchida (1935) reported *Limnesia lembangensis*Piersig, 1906. Since then this group had remained untouched till the 2007’s when Pešić et al. (2008) reported *Pontarachna australis* Smit, 2003.

Water mites of the family Torrenticolidae Piersig, 1902 are presently known from all continents except Antarctica. In general, torrenticolid mites colonize fast flowing streams with well oxygenated interstitial habitats where proto- and tritonymphs can survive the quiescent phase of their life cycle (Di Sabatino et al. 2003).

**Figure 1.**
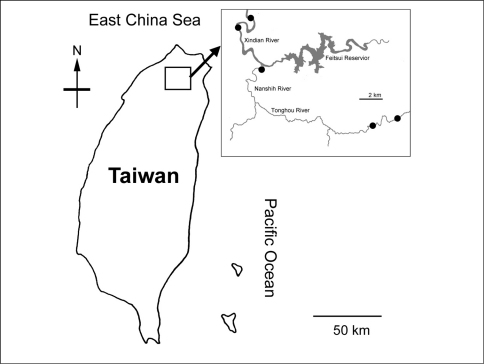
Map of the study area, showing location of sampling sites (black circles).

During a recent survey of the macrozoobenthos of Nanshih River system in Taiwan, some water mites of the genera *Torrenticola* and *Monatractides* were collected. Nanshih River system is located in the northern Taiwan, and originates from Sunglo Lake (1250 m a.s.l.). Drainage network consists of 11 major feeder tributaries (mostly in Wulia District of New Taipei City) connected to Nanshih River which runs into Xindian River in the urbanised Xindian District, the New Taipei City. Collection sites in the present study were located on Xindian River, Nanshih River and one of the tributary Tonghou River (Fig. 1).

Three torrenticolid species are identified, two of them are new to science. Descriptions of these species, as well as the redescription of *Torrenticola ussuriensis* (Sokolow, 1940), that is closely related to one of new species, are given in this paper.

## Material and methods

Water mites were collected by standard Surber sampling method using WaterMark® Surber Type Stream Bottom Sampler (500 μm mesh). All material was preserved in 90% ethanol, and water mites were sorted in the laboratory with the aid of a stereo microscope. The material from Taiwan has been collected by Rita Yam and this is not repeated in the text. Examination of specimens using Scanning Electron Microscopes followed Chatterjee et al. (2009). Holotypes and paratypes of the new species are deposited in the National Museum of Natural Science (NMNS), Taichung, Taiwan. Material of *Torrenticola ussuriensis* (Sokolow, 1940) is deposited in the research collections of the Institute of Biology and Soil Science, Vladivostok, Russia (IBSS).

The composition of the material is given as: males/females/nymphs. All measurements are given in µm. The following abbreviations are used: Cx-1 = first coxae, Cxgl-4 = coxoglandularia of fourth coxae (= E4 in Wiles 1997), Dgl-3 = dorsoglandularia 3, L = length, P-1 = palp, first segment, W = width.

## Systematics

### Family Torrenticolidae. Genus Torrenticola Piersig

#### Torrenticola taiwanicus sp. n.

urn:lsid:zoobank.org:act:9D3C9A70-EF8A-40AB-9680-0257DF27AF91

http://species-id.net/wiki/Torrenticola_taiwanicus

Figs 2 3 4C–D 7A 

##### Type material.

Holotype male (NMNS-6599-001), dissected and slide mounted, Taiwan, Tonghou River, 24°50'23.74"N, 121°38'10.06"E , 25.viii.2009. Paratypes (NMNS-6599-002): 0/2/0 (0/1/0 mounted), Nanshih River, 24°54'09.87"N, 121°33'20.74"E , 02.iii.2010; 0/1/0, ibid., 25.viii. 2009; 1/0/0, Xindian River, 24°56'52.27"N, 121°32'42.54"E , 24.vi.2009; 1/2/0 (1/0/0 mounted), ibid., 16.vii.2009.

##### Diagnosis.

Shoulder plates fused with dorsal plate, the angles of the traces of shoulder plates posterior to setae Dgl-3 weakly pronounced, the angle of dorsal plate between frontal plates slightly pointed, the anterior part of the dorsal plate lying between the traces of the shoulder plates delimitation relatively wide; Cxgl-4 posterior to Cxgl-2, glandular pore Cxgl-4 distanced from Cxgl-2 by 81–90 µm; P-3 distal margin with denticles; P-4 stocky, relatively short (L P-2/P-4 ratio 1.1–1.2), without ventral denticles.

##### Description.

**Figure 2.**
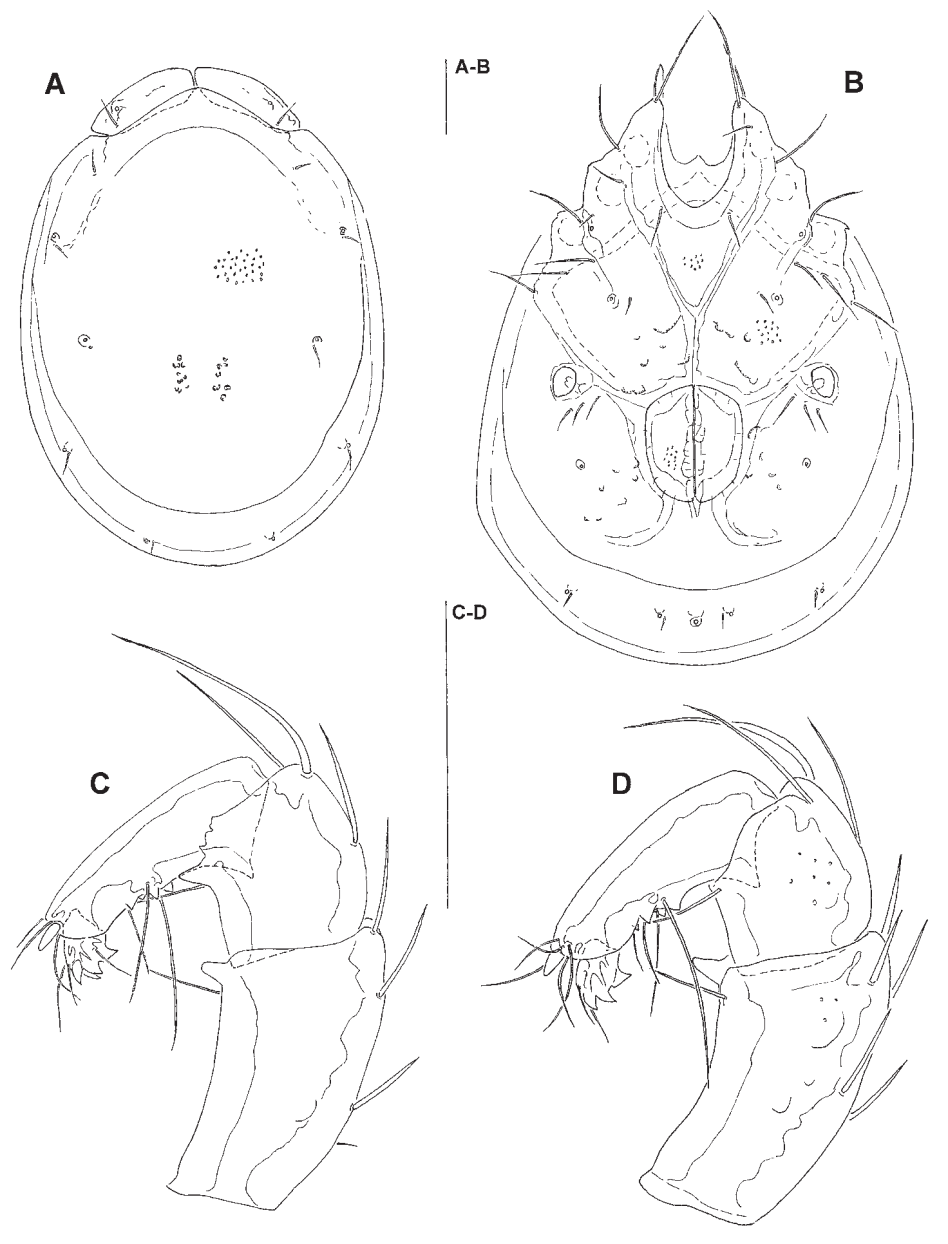
*Torrenticola taiwanicus* sp. n., male: **A** dorsal shield **B** ventral shield **C** palp (P-1 missing), medial view **D** palp (P-1 missing), lateral view. Scale bars = 100 µm.

Male (holotype, in parentheses measurements of paratype). Idiosoma (ventral view: Fig. 2B, 7A) L 741 (734), W 587 (559); dorsal shield (Fig. 2A) L 658 (650), W 488 (494), L/W ratio 1.35 (1.32); dorsal plate L 631 (626); dorsal plate with colour pattern as illustrated in Fig. 4C; frontal plate L 150 (147), W 50 (50–52), L/W ratio 3.0 (2.8–3.0) gnathosomal bay L 139 (131), Cx-1 total L 281 (270), Cx-1 medial L 142 (139), Cx-2+3 medial 85 (91); ratio Cx-1 L/Cx-2+3 medial L 3.3 (3.0); Cx-1 medial L/Cx-2+3 medial L 1.67 (1.53); Cxgl-4 posterior to Cxgl-2, distance between glandular openings of Cxgl-4 and Cxgl-2 81–86 (81–86); genital field L/W 152 (156)/125 (122), L/W ratio 1.22 (1.28), ejaculatory complex conventional in shape, L 234; distance genital field–excretory pore 156 (150), genital field–caudal idiosoma margin 219 (216); capitulum ventral L 322 (328); chelicera total L 378 (383); palp (Figs 2C–D) total L 302 (312), L: P-1 37 (39), P-2 102 (103), P-3 57 (60), P-4 89 (92), P-5 17 (18); P-2/P-4 ratio 1.15 (1.11); distal margin of P-3 with denticles; P-4 with four well developed ventral tubercles.

**Figure 3.**
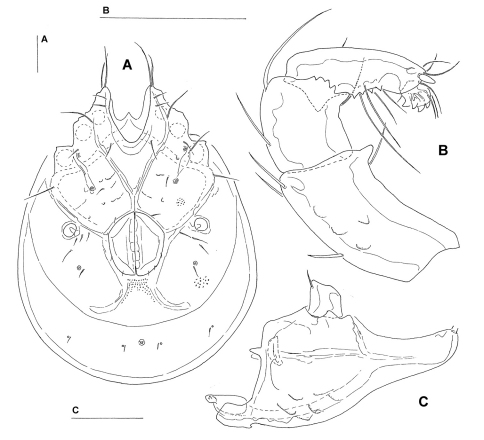
*Torrenticola taiwanicus* sp. n., female: **A** ventral shield **B** palp (P-1 missing), medial view **C** capitulum. Scale bars = 100 µm.

*Female*: Idiosoma (ventral view: Fig. 3A) L 828, W 663; dorsal shield L 756, W 541, L/W ratio 1.4; dorsal plate L 724; frontal plate L 156, W 52–55, L/W ratio 2.8–3.0; gnathosomal bay L 159, Cx-1 total L 300, Cx-1 medial L 141, Cx-2+3 medial 48; ratio Cx-1 L/Cx-2+3 medial L 6.25; Cx-1 medial L/Cx-2+3 medial L 2.9; distance between glandular openings of Cxgl-4 and Cxgl-2 86–90; genital field L/W 170/155, L/W ratio 1.1; distance genital field–excretory pore 181, genital field–caudal idiosoma margin 300; capitulum ventral (Fig. 3C) L 363; palp (Fig. 3B) total L 348, L: P-1 47, P-2 115, P-3 65, P-4 98, P-5 18; P-2/P-4 ratio 1.17; shape and setation as in male.

##### Etymology.

The species is named after the country where it was collected.

##### Remarks.

**Figure 4.**
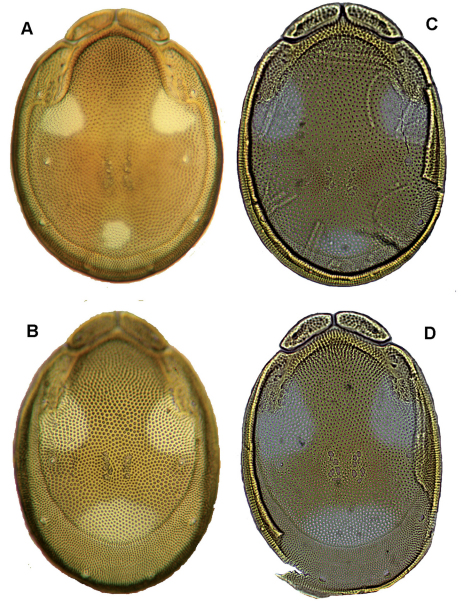
**A–B**: *Torrenticola ussuriensis* (Sokolow, 1940)(**A** male, **B** female), dorsal shield; **C–D**: *Torrenticola taiwanicus* sp. n. (**C** male, **D** female), dorsal shield.

Due to the Cxgl-4 posterior to Cxgl-2 and the shape of palp (distal margin of P-3 with denticles, P-4 stocky and relatively shorter, without ventral denticles), the new species closely resembles *Torrenticola ussuriensis* (Sokolow, 1940) (see below). The latter species differs from *Torrenticola taiwanicus* sp. n., in the shape of dorsal shield (compare Figs 4A–B with Figs 4C–D) with the angle of dorsal plate between the frontal plates more pointed, the anterior part of the dorsal plate lying between the traces of the shoulder plates delimitation more narrower and the angles of the traces of shoulder plates delimitation posterior to setae Dgl-3 more pronounced. A further difference is found in the glandular openings of Cxgl-4 and Cxgl-2 more distanced from each other in *Torrenticola taiwanicus* sp. n. (81–90 vs. 48–60 µm in *Torrenticola ussuriensis*).

*Torrenticola occulta* Lundblad, 1971, a species known from a single juvenile male specimen from Java (Lundblad 1971) resembles *Torrenticola ussuriensis* and *Torrenticola taiwanicus* sp. n., due to the Cxgl-4 posterior to Cxgl-2 and P-4 without ventral denticles, but clearly differs in the shape of palp (see: Lundblad 1971, Fig. 12), with P-2 ventral margin straight and P-4 more slender and relatively longer, L P-2/P-4 ratio 1.0 vs. P-2 ventral margin concave, P-4 more stocky and distinctly shorter than P-2, L P-2/P-4 ratio 1.08–1.2 in *Torrenticola ussuriensis* and *Torrenticola taiwanicus* sp. n. Because some important characters (e.g., the shape and colour of dorsal plate, presence of denticles on distal margin of P-3, distance between the glandular openings of Cxgl-4 and Cxgl-2) were lacking in original description of *Torrenticola occulta*, additional specimens are required to clarify status of this species (see: Wiles 1997 and Pešić and Smit 2009, for an discussion on the Asian *Torrenticola* species that have a dorsal shield with shoulder platelets fused or partially fused with dorsal plate).

##### Distribution.

Taiwan.

#### Torrenticola ussuriensis

(Sokolow, 1940)

http://species-id.net/wiki/Torrenticola_ussuriensis

Figs 4A–B 5 

##### Material examined.

Russia, Primory Territory: Anuchinsky District, 10 km from Vinogradovka, Arsen’evka River, 43°48.261'N, 132°56.407'E , 13.ix.2008, K.A. Semenchenko & D.A. Sidorov 0/1/0; Anuchinsky District, Arsen’evka River near Kornilovka, 43°07.757'N, 133°13.280'E , 03.vi.2009, K.A. Semenchenko & D.A. Sidorov 1/6/0; Khabarovsk Territory, Bikinsky District, Bikin River, 46°46.531'N, 134°17.026'E , 17.vi.2005, K.A. Semenchenko & D.A. Sidorov 0/2/0; Jewish Autonomous Area, Birobidzhansky District, 4 km from Zholty Yar, Bira River, 48°32.373'N, 133°01.664'E , 16.vii.2005, K.A. Semenchenko & D.A. Sidorov 1/2/0; Amurskaya Area, Mazanovsky District, Zeya River, 51°40.034'N, 128°51.265'E , 06.viii.2006, K.A. Semenchenko & T.M. Tiunova 0/2/0.

##### Morphology.

**Figure 5.**
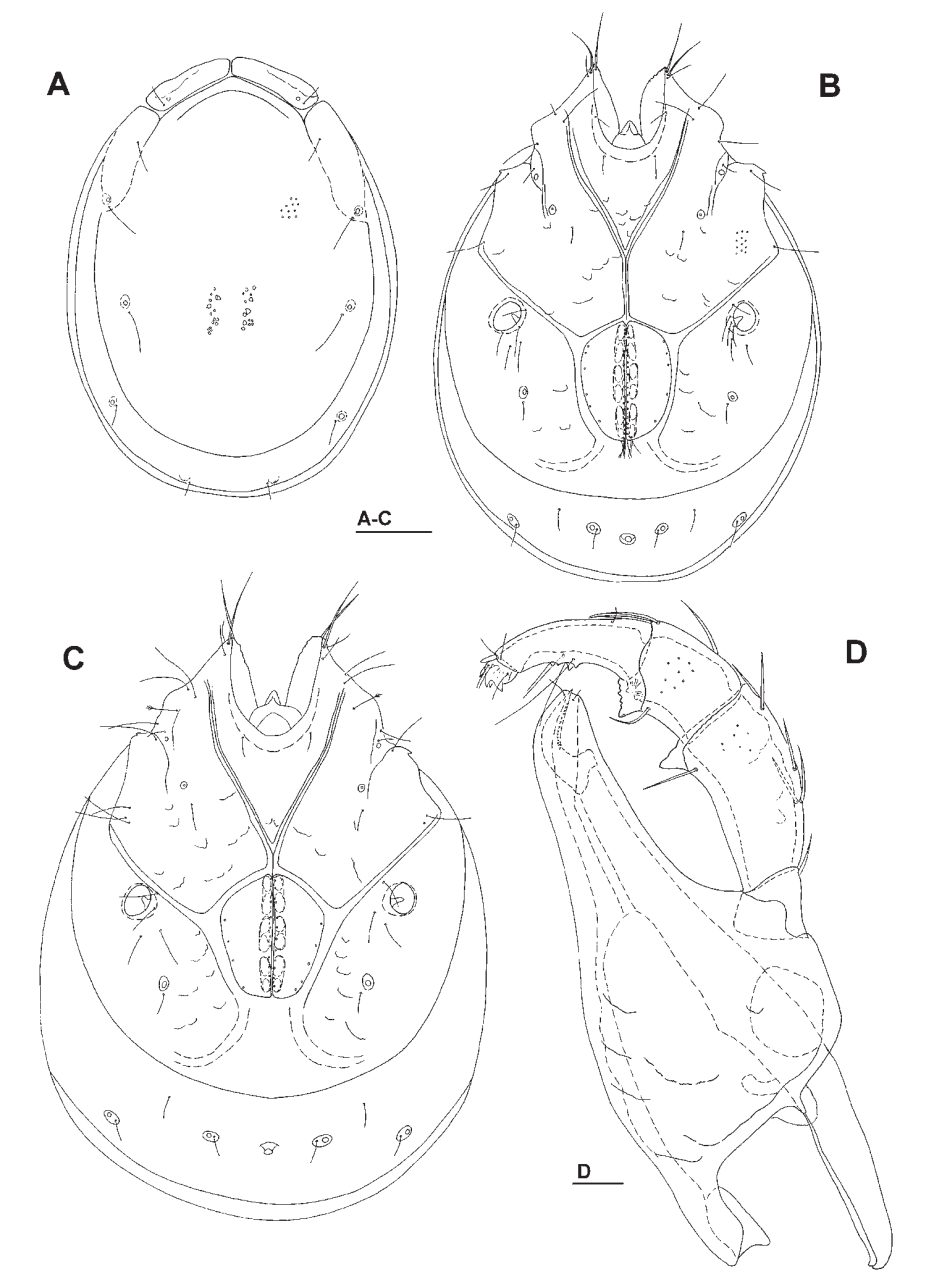
*Torrenticola ussuriensis* (Sokolow, 1940) (**A–B, D** male, **C** female): **A** dorsal shield **B–C** ventral shield **D** capitulum and palp, lateral view. Scale bars = 100 µm (**A–C**), 25 µm (**D**).

*Male* (n = 2). Idiosoma (ventral view: Fig. 5B) L 697–748, W 548–554; dorsal shield (Fig. 5A) L 581–620, W 435–482, L/W ratio 1.28–1.33; dorsal plate L 508–541; dorsal plate with colour pattern as illustrated in Fig. 4A; frontal plate L 119–135, W 44–46, L/W ratio 2.5–3; gnathosomal bay L 110–118, Cx-1 total L 257–264, Cx-1 medial L 152–158, Cx-2+3 medial 72–74; ratio Cx-1 L/Cx-2+3 medial L 3.5; Cx-1 medial L/Cx-2+3 medial L 2–2.19; Cxgl-4 posterior to Cxgl-2, distance between glandular openings of Cxgl-4 and Cxgl-2 48–60; genital field L/W 154–173/118–121, L/W ratio 1.3–1.43, ejaculatory complex conventional in shape, L 153–189; distance genital field–excretory pore 138–165, genital field–caudal idiosoma margin 185–232; capitulum ventral L 302–310; chelicera total L 356–372; palp (Fig. 5D) total L 300–303, L: P-1 32–38, P-2 101–102, P-3 58–59, P-4 89–94, P-5 16–17; P-2/P-4 ratio 1.08–1.12; distal margin of P-3 with denticles; P-4 with four well developed ventral tubercles.

*Female* (n = 2). Idiosoma (ventral view: Fig. 5C) L 782–816, W 594–595; dorsal shield L 643–673, W 468–488, L/W ratio 1.36–1.37; dorsal plate L 547–607; dorsal plate with colour pattern as illustrated in Fig. 4B; frontal plate L 130–132, W 35–40, L/W ratio 3.3–3.7; gnathosomal bay L 125–145, Cx-1 total L 264–284, Cx-1 medial L 140–152, Cx-2+3 medial 46–47; ratio Cx-1 L/Cx-2+3 medial L 5.62–6.16; Cx-1 medial L/Cx-2+3 medial L 3–3.23; distance between glandular openings of Cxgl-4 and Cxgl-2 48–54; genital field L/W 167–172/138–140, L/W ratio 1.19–1.25; distance genital field–excretory pore 191–204, genital field–caudal idiosoma margin 270–363; capitulum ventral L 350–356; chelicera total L 420–435; palp total L 354–358, L: P-1 46–48, P-2 116–119, P-3 69–70, P-4 102–103, P-5 18–21; P-2/P-4 ratio 1.13–1.16; shape and setation as in male.

##### Remarks.

*Torrenticola ussuriensis* was described from the Primory Territory, in Russian Far East (Sokolow 1934) and later reported from Uzi region in Japan (Enami 1940). The specimens from Japan agree with the material from the Far East of Russia in the shape of dorsal shield (e.g., the angle of dorsal plate between the frontal plates pointed, the anterior part of the dorsal plate lying between the traces of the shoulder plates delimitation narrow and the angles of the traces of shoulder plates delimitation posterior to setae Dgl-3 pronounced, see: Enami 1940, Figs 19, 21) and shape of palp (P-3 distal margin with denticles, P-4 relatively short, L P-2/P-4 ratio 1.2 in male, data taken from Enami 1940). However, because some characters (e.g., distance between the glandular openings of Cxgl-4 and Cxgl-2) were lacking in original description, additional specimens are required to confirm that the specimens from Japan are conspecific with *Torrenticola ussuriensis*.

##### Distribution.

Far East of Russia (Primory and Khabarovsk Territory, Jewish Autonomous and Amurskaya Area - Sokolow 1934; present study); Japan (Uzi region - Enami 1940).

#### Torrenticola nanshihensis sp. n.

urn:lsid:zoobank.org:act:6E855396-AE01-45E3-B6A4-59E3D5E3B95B

http://species-id.net/wiki/Torrenticola_nanshihensis

Fig. 6 

##### Type material.

Holotype male (NMNS-6600-001), dissected and slide mounted, Taiwan, Xindian River, 24°56'19.41"N, 121°31'38.38"E , 26.viii.2009.

##### Diagnosis.

Frontal platelets broad (L/W ratio about 1.5); medial suture line Cx-2+3 relatively short; Cx-4 with a prominent suture line of starting at right angle from genital field, laterally curved anteriorly; capitulum deep with a short rostrum; palp robust and compact, P-2 shorter than P-4.

##### Description.

**Figure 6.**
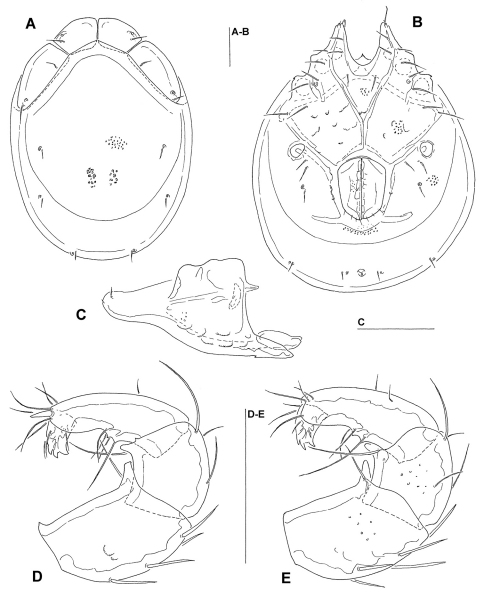
*Torrenticola nanshihensis* sp. n., male: **A** dorsal shield **B** ventral shield **C** gnathosoma **D** palp (P-1 missing), medial view **E** palp (P-1 missing), lateral view. Scale bars = 100 µm.

Male. Idiosoma (ventral view: Fig. 6B) L 700, W 587; dorsal shield (Fig. 6A) L 641, W 481, L/W ratio 1.33; dorsal plate L 578; frontal plate L 123–125, W 81–84, L/W ratio 1.49–1.52; shoulder plate L 184, W 83, L/W ratio 2.2; L shoulder/frontal plate ratio 1.47; gnathosomal bay L 109, Cx-1 total L 253, Cx-1 medial L 142, Cx-2+3 medial 94; ratio Cx-1 L/Cx-2+3 medial L 2.7; Cx-1 medial L/Cx-2+3 medial L 1.5; genital field L/W 159/123, L/W ratio 1.3, ejaculatory complex conventional in shape, L 256; distance genital field–excretory pore 141, genital field–caudal idiosoma margin 194; capitulum (Fig. 6C) ventral L 269; chelicera total L 290; palp (Figs 6D–E) total L 303, L: P-1 34, P-2 89, P-3 62, P-4 93, P-5 25; L P-2/P-4 ratio 0.96; P-4 with well developed ventral tubercles.

##### Etymology.

The species is named after Nanshih River system where it was collected.

##### Remarks.

Due to the deep capitulum with a short rostrum, a prominent suture line of Cx-4 starting at right angle from genital field, a relatively short medial suture line of Cx-2+3 and the similar shape of the palp with robust and compact segments, P-2 shorter than P-4, and P-4 with well developed ventral tubercles, the new species resembles *Torrenticola brevirostris* (Halbert, 1911). *Torrenticola nanshihensis* sp. n., can be easily distinguished from the latter species in having broad frontal platelets (L/W ratio about 1.5).

##### Disribution.

Taiwan; known only from the *locus typicus*.

### Genus Monatractides K. Viets

#### Monatractides circuloides

(Halík, 1930) cf.

http://species-id.net/wiki/Monatractides_circuloides

Fig. 7B–D 

##### Material examined.

Taiwan, Tonghou River, 24°50'23.74"N, 121°38'10.06"E , 25.viii.2009, 2/0/0 (1/0/0 mounted); ibid., 24°50'23.74"N, 121°38'26.52"E , 15.vii.2009 3/0/0; ibid., 25.viii.2009 1/1/0; ibid., Nanshih River, 24°54'09.87"N, 121°33'20.74"E , 02.iii.2010, 0/1/0 (0/1/0 mounted); ibid., 25.viii.2009, 0/1/0; Xindian River, 24°56'52.27"N, 121°32'42.54"E , 24.vi.2009 2/0/0; ibid., 24°56'19.41"N, 121°31'38.38"E , 26.viii.2009 2/0/0.

##### Morphology.

*Male*. Idiosoma L 966, W 719; dorsal shield L 806, W 600, L/W ratio 1.34; dorsal plate L 754; shoulder plate L 219, W 84, L/W ratio 2.6; frontal plate L 147, W 75, L/W ratio 1.85; shoulder/frontal plate L ratio 1.5; capitular bay L 188, its lateral margin with the three pairs of knob-shaped protrusions; Cx-1 total L 326, Cx-1 medial L 138, Cx-2+3 medial 127; ratio Cx-1 L/Cx-2+3 medial L 2.57; Cx-1 medial L/Cx-2+3 medial L 1.09; genital field L/W 188/144, L/W ratio 1.3; ejaculatory complex L 259; distance genital field–excretory pore 227, genital field–caudal idiosoma margin 320. Capitulum ventral L 220; chelicera L 262; palp total L 254, dL: P-1 29, P-2 83, P-3 47, P-4 63, P-5 32; P-2/P-4 ratio 1.3; P-4 with well visible denticle near the insertion of the ventral hairs; L I-L-4–6: 127, 120, 123.

*Female*. Idiosoma L 1094, W 806; dorsal shield L 894, W 687, L/W ratio1.3; dorsal plate L 851; shoulder plate L 213, W 84, L/W ratio 2.54; frontal plate L 150, W 72, L/W ratio 2.08; shoulder/frontal plate L ratio 1.42; capitular bay L 205; Cx-1 total L 328, Cx-1 medial L 123, Cx-2+3 medial 105; ratio Cx-1 L/Cx-2+3 medial L 3.12; Cx-1 medial L/Cx-2+3 medial L 1.17; genital field L/W 206/194, L/W ratio 1.06; distance genital field–excretory pore 264, genital field–caudal idiosoma margin 438; capitulum ventral L 238; chelicera L 258; palp total L 260, L: P-1 29, P-2 85, P-3 50, P-4 64, P-5 32; P-2/P-4 ratio 1.33; L I-L-4–6: 135, 126, 120.

**Figure:**
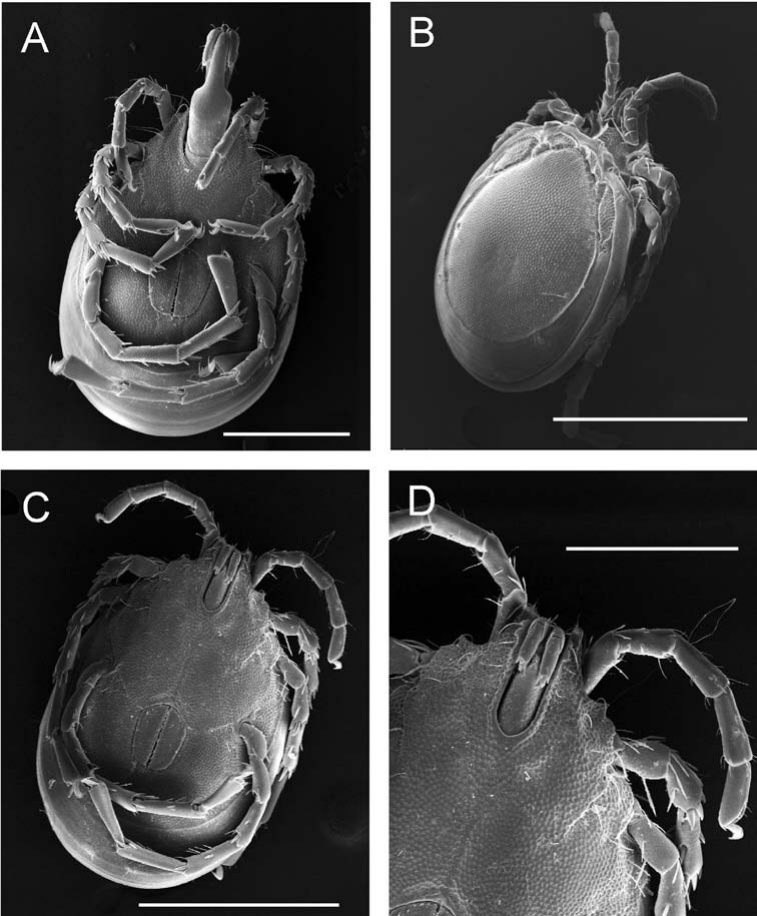
**Figure 7.** SEM photographs. **A** *Torrenticola taiwanicus* sp. n., female: **A** ventral view of idiosoma and gnathosoma, scale bar = 200 μm. **B–D** *Monatractides* cf. *circuloides* (Halík, 1930), male: **B** dorso-lateral view of idiosoma and gnathosoma, scale bar = 500 μm **C** ventral view of idiosoma and gnathosoma, scale bar = 500 μm. **D** ventral view of gnathosoma and anterior part of idiosoma, scale bar = 200 μm.

##### Remarks.

Due to the presence of three pairs of knob-shaped protrusions at the margin of the capitular bay (Fig. 7D), a rounded capitular bay (Fig. 7D), a short capitular rostrum, relatively longer median suture line of Cx-2+3 (Fig. 7C–D), and the posterior medial region behind the genital field pointed, the specimens from Taiwan agree well with *Monatractides circuloides* (Halík, 1930), a species known from Malaysia and Thailand (Pešić and Smit 2009, 2010) Differences (in parentheses measurements of male specimen from Malaysia, data taken from Pešić and Smit 2010) are found in its larger idiosoma and palp dimensions (e.g., idiosoma L 881, dorsal shield L 763, genital field L/W 170/134, P-2 L 69, P-4 L 54). Knowledge on the degree of variability of the additional specimens from a wide area is necessary before we can assess the taxonomic status of these populations.

##### Distribution.

Malaysia, Thailand, Taiwan.

## Supplementary Material

XML Treatment for Torrenticola taiwanicus

XML Treatment for Torrenticola ussuriensis

XML Treatment for Torrenticola nanshihensis

XML Treatment for Monatractides circuloides
